# Do diets with higher inflammatory potential increase the risk of dementia in people aged 50 and over?

**DOI:** 10.1002/alz70860_102773

**Published:** 2025-12-23

**Authors:** Natalia Cochar‐Soares, Sara Souza Lima, Thaís Barros Pereira da Silva, Sherry Price, James R. Hebert, Andrew Steptoe, Cesar de Oliveira, Tiago da Silva Alexandre

**Affiliations:** ^1^ Federal University of São Carlos, São Carlos, São Paulo, Brazil; ^2^ Federal University of Sao Carlos, São Carlos, São Paulo, Brazil; ^3^ University of South Carolina, Columbia, SC, USA; ^4^ University College London, London, England, United Kingdom; ^5^ University College London, London, Greater London, United Kingdom

## Abstract

**Background:**

Recent studies have shown that almost half of all dementia cases could have been prevented or delayed by managing modifiable risk factors. However, there is a paucity of results from longitudinal studies that have analysed the association between the inflammatory potential of the diet, assessed by the Dietary Inflammatory Index (DII^®^), and the risk of dementia. We aimed to analyse whether a diet with a higher inflammatory potential is a risk factor for the incidence of dementia in a three‐year follow‐up.

**Method:**

Data from a total of 2,346 participants from the *English Longitudinal Study of Ageing (ELSA Study)* aged 50 years or older were included. The DII was calculated from dietary intake data collected using the Oxford WebQ questionnaire and the energy‐adjusted Dietary Inflammatory Index (E‐DII™) to exclude the effect of energy intake on nutrients. The E‐DII score was divided into tertiles, where the lowest tertile (≤ ‐0.021) represents a diet with lower inflammatory potential, the second tertile (> ‐0.021 and ≤ 1.664) represents a medium inflammatory potential, and the highest tertile (> 1.664) represents the highest inflammatory potential of the diet. The incidence of dementia was defined as the presence of physician‐diagnosed dementia or the coexistence of cognitive impairment (global cognition z‐score lower than 1.5 standard deviations compared to the sample mean) and functional impairment (difficulty in performing one or more basic activities of daily living). Poisson regression models were performed to analyse the incidence of dementia, adjusted for socioeconomic, behavioural, anthropometric and clinical variables.

**Result:**

Participants with a higher dietary inflammatory potential, classified in the second and third tertiles, had a higher risk of dementia incidence over the three years of follow‐up (IRR = 1.96, 95%CI 1.08 – 3.54, and IRR = 2.08, 95%CI 1.16 – 3.74, respectively) when compared to individuals classified in the first tertile.

**Conclusion:**

The greater the inflammatory potential of the diet, the greater the risk of dementia in people aged 50 years or over. Therefore, given that diet is a modifiable factor, it can be adjusted to reduce its inflammatory potential and, consequently, reduce the risk of dementia.

